# Advances in pervaporation desalination based on polymer membranes

**DOI:** 10.1039/d5ra00895f

**Published:** 2025-06-20

**Authors:** Yufang Xu, Chuanying Wang, Qinghui Ling, Lei Sang

**Affiliations:** a Department of Pharmacy, Anhui Medical College Hefei China sanglei0114@163.com

## Abstract

Desalination is the process of removing salts and minerals from saline water to produce potable water. It is a critical global challenge due to the increasing demand for freshwater. Pervaporation (PV) is a membrane-based separation process that combines sorption and permeation, and it has emerged as a promising alternative to traditional desalination methods. This review provides a comprehensive overview of recent advancements in the development and application of polymer membranes for PV desalination. We begin by discussing the fundamental principles of PV and exploring its mechanism, underscoring its preparation methods, such as solution coating, solution casting, and interfacial polymerization. The review then delves into various types of polymer membranes used in PV desalination, such as cellulose and its derivatives, polyvinyl alcohol, polyacrylonitrile, polyamides and sulfonated block copolymers, describing their chemical structures, synthetic techniques, and performance characteristics. Special attention is given to the role of membrane properties—such as hydrophilicity, compositions and functionality—in determining the efficiency of salt rejection and water flux. Then, the cleaning of contaminated PV polymer-based membranes is reviewed. Furthermore, we discuss the challenges and limitations associated with polymer membranes in PV desalination, which include fouling, swelling, and chemical degradation, and present strategies to mitigate these issues. The review aims to serve as a resource for researchers, engineers, and policymakers interested in advancing the state of the art in PV desalination technologies and addressing the global water scarcity crisis through innovative membrane science.

## Abstract 2

Desalination is the process of removing salts and minerals from saline water to produce potable water.

## Graphical abstract

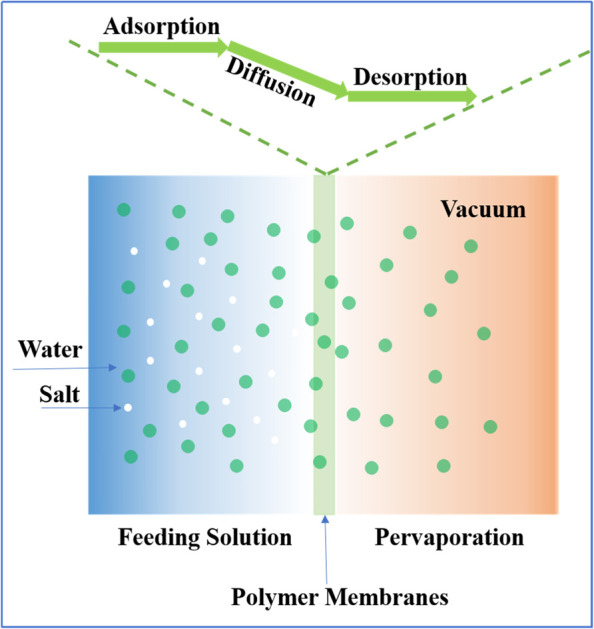

## 1. Introduction

As the human population expands, water scarcity impacts more than half of the world's inhabitants, with approximately 663 million lacking access to potable water.^1^ The escalating demand for water in agriculture and industry is rapidly increasing global freshwater consumption. Given that 97% of the Earth's water is saline and unusable for most purposes, and only a fraction over 1% is directly accessible as freshwater, desalination emerges as a crucial solution to bolster freshwater availability and ensure global water security.^2^ Although desalination has seen substantial progress in recent decades, there remains ample opportunity for further development, both in refining current methods and pioneering novel desalination strategies.

Membrane separation is a low-energy desalination technology that has been industrially applied in processes such as seawater desalination, resource utilization of industrial saline wastewater, and deep purification of sewage, mainly involving pressure-driven membrane, electrically driven membrane, and thermal membrane desalination methods.^3–10^ Compared with the first two technologies, thermal membrane desalination technology is less affected by the osmotic pressure and polarization effects of the feed solution. Currently, thermal membrane desalination mainly includes membrane distillation (MD) and pervaporation (PV). MD requires more complex pretreatment conditions during the desalination process, and its hydrophobic membrane surface is prone to contamination or crystallization, leading to surface wetting and membrane leakage issues.^11–13^ PV membranes combine permeation and vaporization, where a liquid feed mixture is brought into contact with one side of a semi-permeable membrane, and a vacuum or sweep gas is applied on the permeate side to create a chemical potential difference for separation.^14–17^ Generally, PV membranes have a dense hydrophilic selective separation layer that can effectively avoid leakage problems caused by membrane contamination and crystallization during the desalination process.

In recent years, the interest in PV desalination technology has been growing with the development of PV membranes that possess high water flux and a high salt rejection rate. However, PV desalination technology still faces issues such as low mechanical strength, susceptibility to swelling, poor long-term stability, and low permeation flux in high-concentration brine solutions.^18–20^ Based on the above content, this review starts with an exploration of the mechanism of PV, underscoring its preparation methods, *e.g.*, solution coating, solution casting, and interfacial polymerization (IP). Subsequently, the review scrutinizes the diverse polymeric membranes employed in PV desalination, such as cellulose and its derivatives, polyvinyl alcohol (PVA), polyacrylonitrile (PAN), polyamides (PA) and sulfonated block copolymers, outlining their chemical configurations, synthesis techniques, and performance. Emphasis is placed on the influence of membrane attributes—ranging from hydrophilicity, composition to functionality—on the effectiveness of salt rejection and water flux. Then, the cleaning of contaminated PV polymer-based membranes was reviewed. Finally, we discuss the challenges and limitations associated with polymer membranes in PV desalination, such as fouling, swelling, and chemical degradation, and present strategies to mitigate these issues.

## 2. Desalination mechanism of PV and preparation methods of polymer membranes

### 2.1. Desalination mechanism of PV membranes

The very nature of PV integrates the processes of permeation and evaporation within a membrane framework. On the feed side, the membrane interfaces with the solution, selectively allowing certain components to permeate while others are retained. The feed side operates at atmospheric pressure, contrasting with the vacuum or swept gas conditions on the outlet side, which facilitates the transition of the permeated liquids into vapor.^21^ After passing through the membrane, a cold trap at the downstream end quickly condenses or traps these vapors and collects them inside. After the purified permeate is condensed, it is collected and analyzed.

**Fig. 1:**
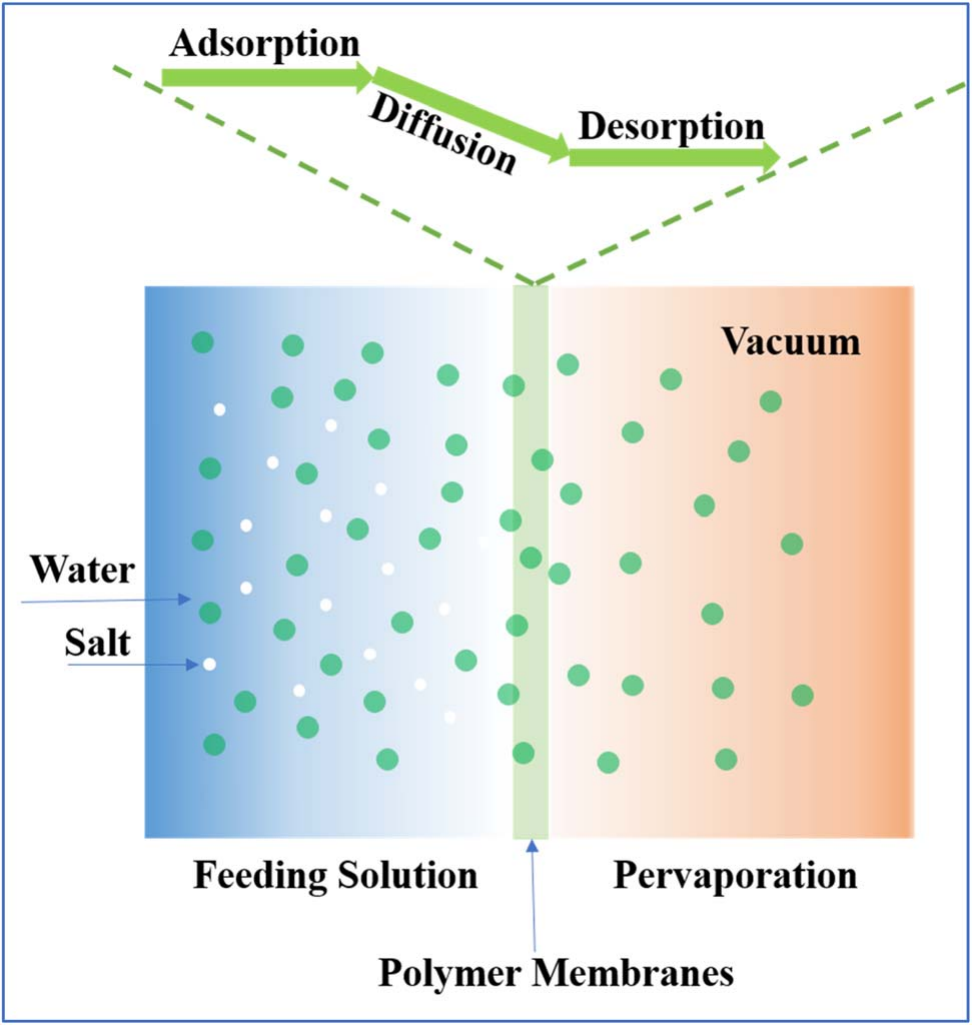
Illustration of the water transportation mechanism in PV desalination membranes.

The transport across the membrane is driven by a solution–diffusion mechanism, with the chemical potential gradient across the membrane serving as the impetus (Fig. 1). Each pervaporation membrane exhibits unique selectivity for various components, resulting in distinct diffusion rates for each, enabling the membrane to effectively separate the feed components, yielding a purified permeate and a concentrated waste stream.^22–24^ The continuous operation is facilitated by the vacuum pump, which maintains the chemical potential difference for the desired components, ensuring sustained transport throughout the process. The performance of PV membranes is commonly evaluated from two main aspects, *i.e.*, permeability and separation property, in which water flux and the salt rejection rate are used to quantitatively determine performance.^25,26^

The performance of PV desalination using membranes is influenced by a variety of parameters, such as the roles of charge interactions, pore size, and selective transport.^18,21^ Electrostatic interactions primarily achieve selective separation through the electrostatic repulsion/attraction effects between the charge density of the membrane surface or pores and ions. When the membrane surface carries a negative charge, it can effectively retain anions (such as Cl^−^ and SO_4_^2−^) through electrostatic repulsion. For example, a PVA membrane modified with negatively charged sulfonic acid groups (−SO_3_H), known as S-PVA, has a near 100% rejection rate for SO_4_^2−^, while the rejection rates for Mg^2+^ and Na^+^ are lower (∼60% and 50%, respectively). This selectivity is related to the ionic valence and hydration radius and the strong repulsion between multivalent anions and the negative charges on the membrane surface is the origin of this selectivity.^27^ Pore size directly affects the molecular sieving effect and mass transfer resistance, and a balance between flux and selectivity is required.^22^ The ideal pore size of a PV membrane (0.3–1 nm) should be smaller than the diameter of hydrated ions (*e.g.*, the hydration radius of Na^+^ is approximately 0.36 nm, and that of Cl^−^ is about 0.33 nm), but it must allow water molecules (with a diameter of approximately 0.28 nm) to pass through rapidly. Selective transport relies on the synergistic action of the adsorption–diffusion mechanism and the chemical potential gradient. Water molecules preferentially adsorb onto the hydrophilic membrane surface and enter the membrane through Fickian diffusion or facilitated diffusion.^22,28^ For example, sulfonic acid groups in sulfonated PVA membranes reduce the diffusion energy barrier for water molecules through long-range interactions, increasing the water diffusion coefficient in the dry membrane region, which overcomes the traditional trade-off between flux and selectivity.

### 2.2 Preparation methods

#### 2.2.1. Solution coating

The solution coating technique is extensively utilized for fabricating PV polymer membranes, especially for developing composite membranes that exhibit superior performance.^29–31^ Typically, a thin, selective layer is applied to a microporous support, which may be flat, hollow-fiber, or tubular. The support must be fully porous to reduce structural resistance, ensuring that the membrane's resistance is predominantly determined by the selective layer. The process starts with the preparation of a coating solution, usually consisting of a polymer dissolved in an appropriate solvent. Before applying the coating solution, the support is pre-wetted with a low boiling point solvent that is immiscible with the coating solvent to prevent intrusion. Then, techniques such as spray coating or dip coating are used to apply the solution to the support.^32–34^ Subsequently, the coated support is dried to evaporate the solvent, resulting in a uniform polymer membrane.

A significant advantage of the solution coating method is its capacity to produce membranes with controlled thickness and uniformity, which are essential for achieving high selectivity and permeation rates in pervaporation desalination.^35^ For instance, PVA solutions have been effectively coated onto PAN supports to create composite membranes with enhanced desalination performance. This method also permits the addition of additives or fillers to the coating solution, further improving the membrane's properties, such as hydrophilicity and mechanical strength. Lee *et al.* reported the fabrication of robust composite membranes using graphene quantum dots and two kinds of cationic polyelectrolytes by the solution coating method with porous PAN substrate.^36^ Remarkably, a 925 g m^−2^ h^−1^ permeation flux was achieved while exhibiting a 98 wt% water concentration permeation for the composite membranes.

However, the solution coating method necessitates meticulous control over the coating conditions to ensure uniform film formation and prevent defects. Additionally, the selection of the support and coating solution must be carefully optimized to achieve the desired performance characteristics for pervaporation desalination applications.

#### 2.2.2. Solution casting

The solution casting method is a widely employed technique for the fabrication of polymer membranes used in separation, antifouling and antibacterial processes.^37–39^ It is favored for its simplicity, versatility, and ability to produce membranes with controlled thickness and uniformity. The method involves dissolving a polymer in a suitable solvent to create a homogeneous solution and casting it onto a flat substrate, typically a glass plate or a similar surface. Subsequently, the solvent is evaporated, leaving behind a uniform polymer film. Li *et al.* fabricated two types of lotus-inspired polydimethylsiloxane (PDMS) composite membranes using the solution casting method: lotus leaf powder/PDMS mixed matrix membranes (MMMs) and polydivinylbenzene (PDVB)-coated PDMS composite membranes.^40^ Since PDVB exhibits preferential adsorption for ethanol, the PDVB-coated PDMS membrane achieves a higher ethanol recovery, with the separation factor and total flux increasing by 13% and 30%, respectively. This demonstrates that PDVB coating is an effective technique for creating superhydrophobic membranes, and both lotus-inspired strategies are viable for enhancing pervaporation performance in ethanol recovery. Additionally, Mohammadi *et al.* developed PVA/zeolite 4A mixed matrix composite membranes supported on polypropylene microfiltration membranes using the solution casting method and crosslinked with glutaraldehyde to study their pervaporation separation properties for water–ethylene glycol mixtures.^41^ In a separate study, Flynn *et al.* integrated spherical, discrete, size-monodisperse mesoporous silica particles—measuring 1.8–2 μm in diameter and with pores of approximately 1.8 nm—into a PVA matrix to develop composite pervaporation membranes. These membranes' selective layers were cast onto supports made of polyacrylonitrile (PAN) and non-woven fabric.^42^

Despite its benefits, the solution casting method presents challenges such as the need for precise control over the casting conditions to ensure uniform film formation and the potential for defects in the membrane structure. However, these issues can be mitigated with advancements in materials science and process optimization, making solution casting a robust approach for producing high-performance polymer membranes in PV desalination applications.

#### 2.2.3. Interfacial polymerization

IP enables the attainment of high permeate flux without compromising membrane selectivity, thus challenging the conventional trade-off between permeate flux and separation selectivity. Owing to its reproducibility and scalability, IP has found extensive application in the realms of nanofiltration and reverse osmosis.^43–45^ However, the preparation of thin-film composite (TFC) membranes for PV using IP has not been widely reported. Since IP can be controlled to produce thinner and dense defect-free layers to meet the requirements of PV membranes, this technique holds broad application potential in the field of PV.^46–48^ IP generally involves the use of two highly reactive monomers that undergo a polymerization reaction at the interface of immiscible solvents, thereby forming a dense layer on a porous support. Upon contact between the aqueous-phase reactant and the organic-phase reactant, a rapid reaction ensues, leading to the immediate formation of a thin film at the interface. Due to the low concentration of reactants at the interface, and the need for unreacted monomers to pass through the formed film to come into contact and continue the reaction, the reaction rate is reduced, allowing for the formation of an extremely thin separation-selective layer.^49^ It has been reported that the selective layer in IP can be as thin as 20 nm. The support is immersed in an aqueous solution containing a reactive monomer, allowing it to become fully saturated, and the excess solution is drained off. Then, the membrane is immersed in an organic (oil) phase containing another reactive monomer and the two reactive monomers react with each other on the surface of the support to form a dense polymer layer.

The characteristics of IP reactions are as follows: (i) the morphology and thickness of the active layer can be well controlled by adjusting parameters such as the concentration of the monomers in the two phases, temperature, and reaction time;^50^ (ii) the morphology of the base membrane can regulate the solubility and diffusion rate of the amine in the reaction zone, thereby determining the performance and structural characteristics of the IP membrane;^51^ (iii) the reaction typically occurs on the organic phase side, with the aqueous-phase monomer needing to diffuse to the interface of the oil-phase monomer to react;^52^ (iv) the adhesion between the support layer and the active layer can be increased by selecting an appropriate base membrane or modifying the base membrane, thereby enhancing the stability of the membrane.^53^ Therefore, current research in this field focuses on selecting or modifying an appropriate base membrane as well as optimizing monomer structure and polymerization parameters to produce high performance PV desalination membranes.

**Fig. 2:**
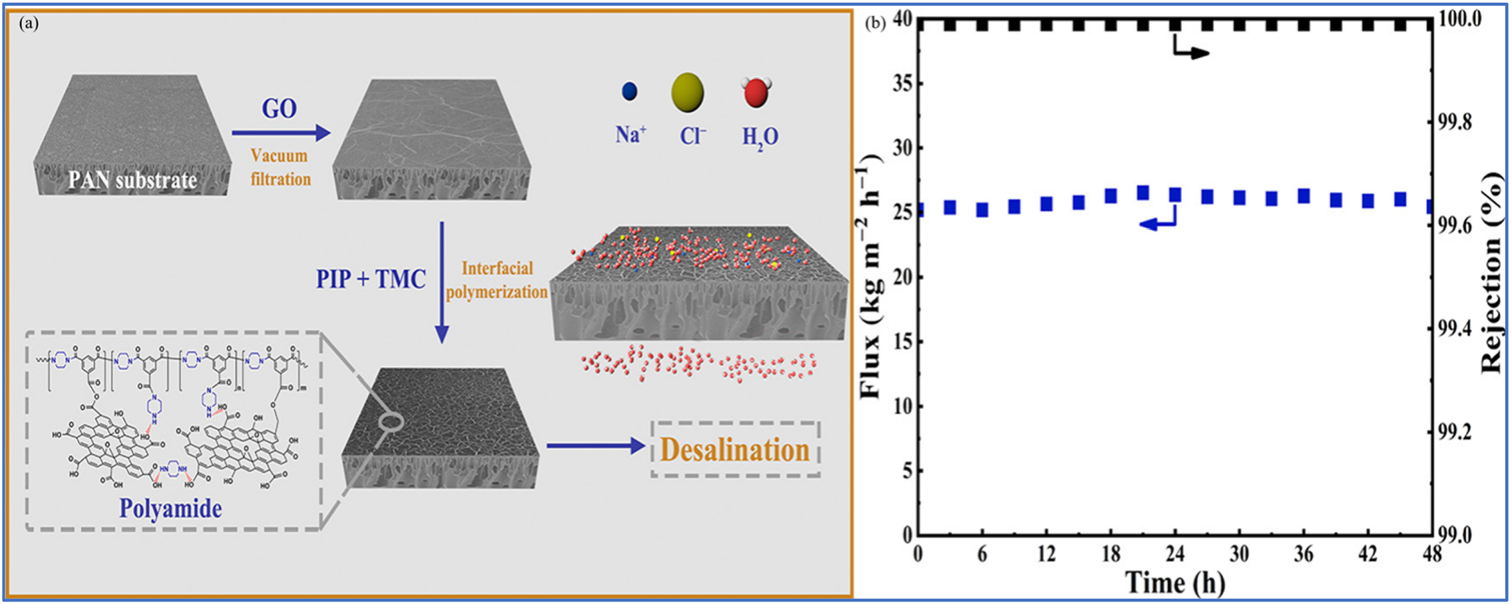
(a) Illustration of the preparation process of PAG@GO-PA PV composite membranes. (b) Long-term water flux and salt rejection of PAG@GO-PA PV (3.5 wt% NaCl).^54^ Reproduced from ref. 54 with permission from American Chemical Society, copyright 2020.

Zhang *et al.* reported a method that initially involved vacuum filtration to introduce graphene oxide (GO) with hydroxyl and carboxyl groups on its surface onto a PAN substrate, serving as a functionalized intermediate layer (Fig. 2(a)).^54^ Subsequently, the PAN@GO composite was immersed in a piperazine aqueous solution, allowing piperazine (PIP) molecules to intercalate into the GO layers or graft onto the GO surface. Finally, a dense PA film was formed on the GO surface through the IP reaction between piperazine and benzenetricarbonyl trichloride (TMC). Additionally, the hydroxyl groups on the GO surface participated in the reaction with TMC, forming a crosslinked network structure that effectively suppressed the swelling of GO and increased the water molecule channels. The study found that the composite membrane maintained a long-term salt rejection rate of 99.99% when treating a 3.5 wt% NaCl aqueous solution, with the water flux reaching 26.7 kg m^−2^ h^−1^ (Fig. 2(b)).

It is generally believed that the permeate flux of a membrane is negatively correlated with its thickness.^55^ Thus, a decreased thickness of the PA-based PV desalination membranes is beneficial to its water flux and salt rejection performance. Having this in mind, Niu *et al.* found that the thickness of the PA membranes is reduced by about 50 nm when using an organic phase monomer containing 5-sulfinyl amino isophthaloyl dichloride (NSO) in the IP with PIP compared with the separation layer formed by the IP of PIP and TMC.^56^ This is because of the dual roles of NSO in the IP process: it binds with H^+^ ions, thereby acting as an acid scavenger to facilitate the IP process; it can also transform into an amino group, which allows it to further engage in the IP reaction, enhancing the cross-linking density of the PA layer. By finely optimizing the reaction parameters, the produced PIP-NSO PV membrane achieved a water flux of 50.54 kg m^−2^ h^−1^, alongside a salt rejection rate of 99.99% with a 3.5 wt% NaCl aqueous solution at 70 °C.

## 3. Polymer membranes in PV desalination

### 3.1. PVA

**Fig. 3:**
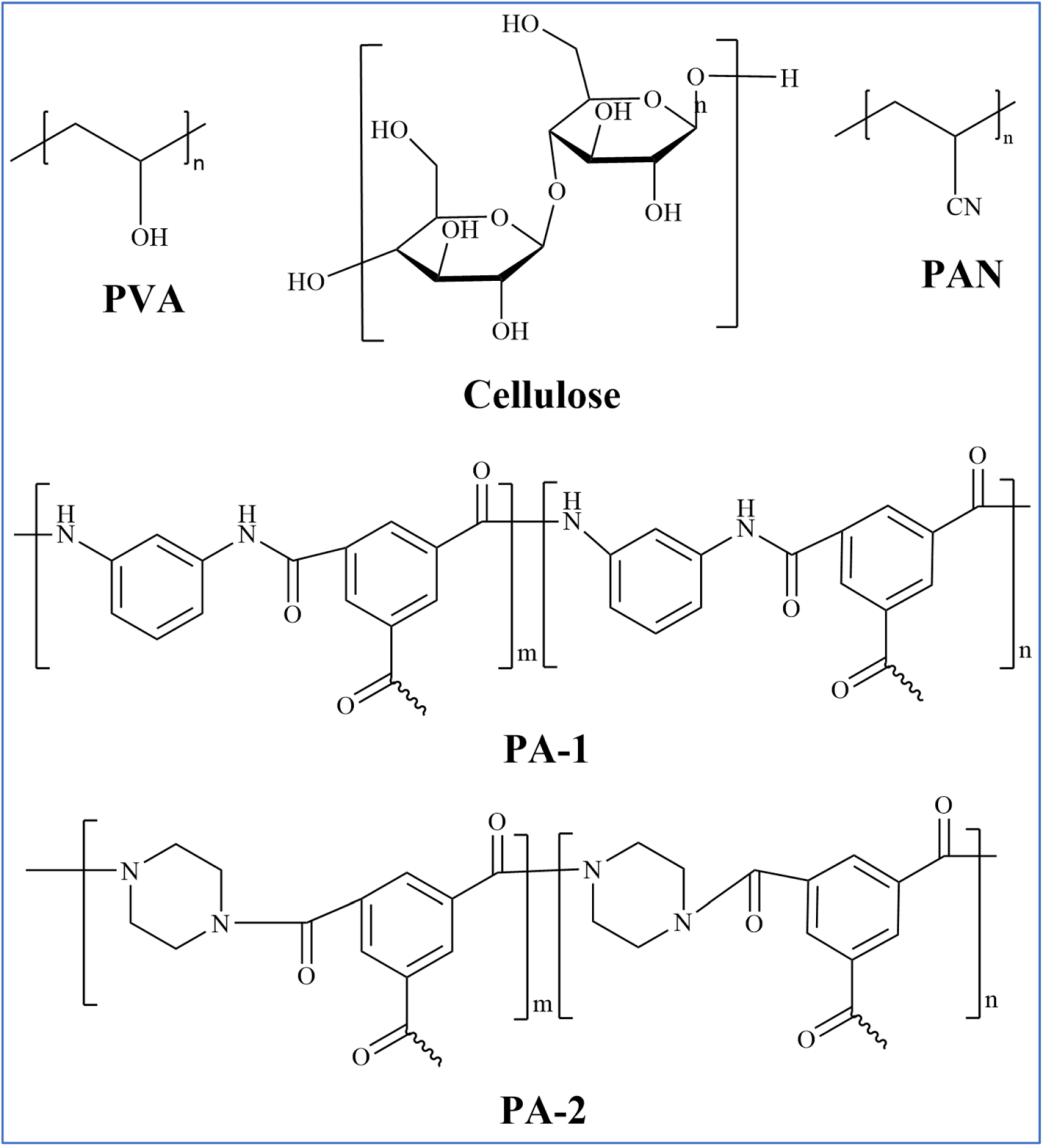
Chemical structures of different polymers in PV membranes.

**Fig. 4:**
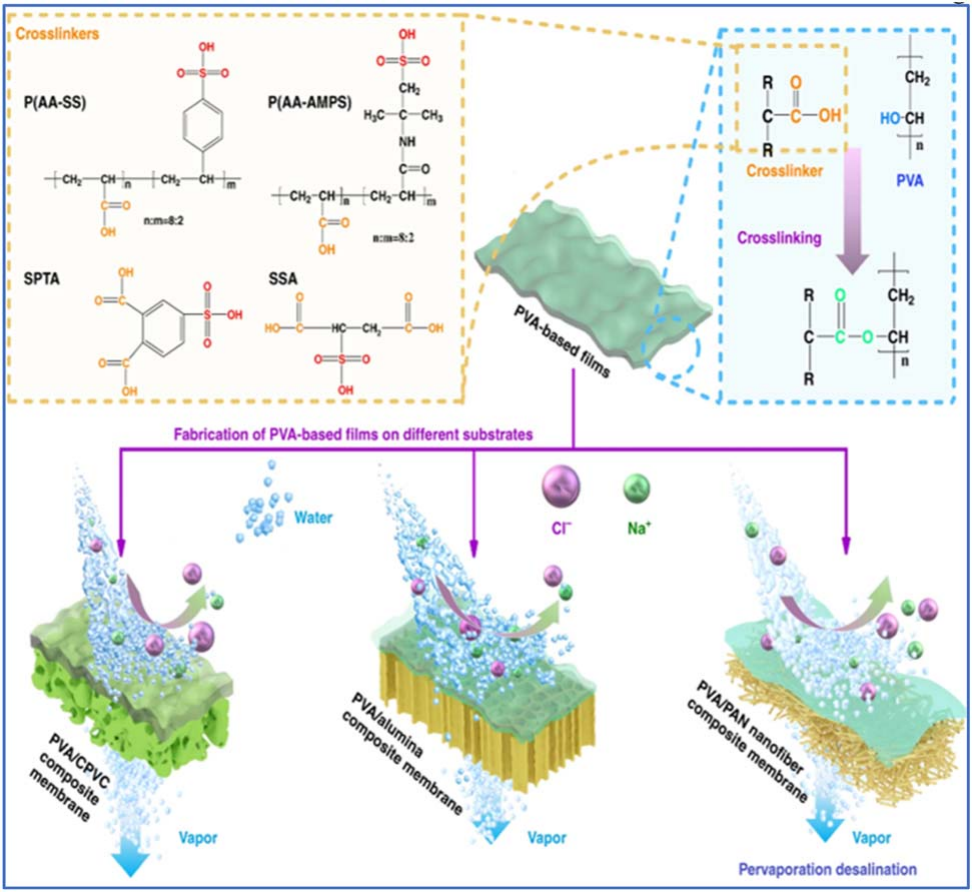
Illustration of the chemical structures of crosslinkers, crosslinking reaction details and water transport in PV membranes.^62^ Reproduced from ref. 62 with permission from Elsevier, copyright 2022.

**Fig. 5:**
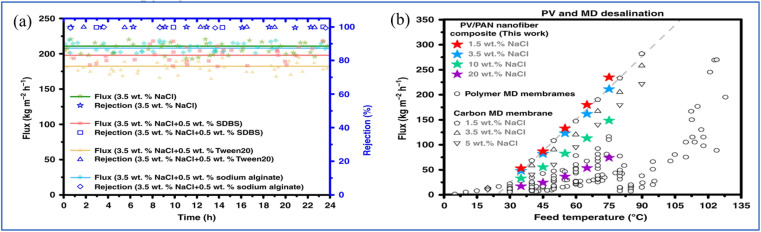
(a) Water flux and salt rejection of PVA-based composite membrane (3.5 wt% NaCl). (b) Comparing the water flux in this work with other reported publications.^62^ Reproduced from ref. 62 with permission from *Nature*, copyright 2020.

PVA is a white powdery crystalline polymer obtained from the hydrolysis of polyvinyl acetate, featuring a large number of hydroxyl groups in its side chains (Fig. 3) which form the basis for its chemical modification and cross-linking.^57^ PVA is renowned for its exceptional hydrophilicity, superior film-forming capabilities, resistance to organic contamination, non-toxicity, biodegradability, and robust chemical stability, making it the predominant material for PV film applications.^58–60^ PVA possesses excellent hydrophilicity (capable of effectively adsorbing and transporting water molecules to enhance water permeability), tunability of membrane structure and functionality (through chemical modifications such as sulfonation or copolymerization, or by forming composites with other materials like nanofillers), and environmental friendliness (*e.g.*, using water as a solvent and biodegradability). However, its solubility in water necessitates cross-linking to enhance stability and mechanical performance, which is typically achieved through esterification or acetal reactions. Monomer cross-linking agents, such as sulfonated succinic acid (SSA) and sulfosalicylic acid (SPTA), are known to form carboxyl dimers characterized by strong hydrogen bonding.^61^ In contrast, long-chain asymmetric crosslinking agents, including poly(acrylic acid-*co*-2-acrylanmido-2-methylpropanesulfonic acid) (P(AA-AMPS)) and poly(acrylic acid-*co*-sulfonated styrene) (P(AASS)), are more prone to forming ester groups. Li *et al.* employed molecular dynamics simulations to optimize the selection of crosslinking agents for PVA-based PV membranes, evaluating various agents such as sulfonated succinic acid (SSA), sulfosalicylic acid (SPTA), and poly(AA-AMPS) (P(AASS)) (Fig. 4).^62^ Their findings revealed that long-chain crosslinking agents exhibited superior crosslinking efficiency compared with their short-chain counterparts. Specifically, a PVA/PAN nanofiber composite membrane crosslinked with P(AA-AMPS) was tested in pervaporation experiments at 75 °C using a 1.5 wt% NaCl solution, achieving a water flux of 234.9 ± 8.1 kg m^−2^ h^−1^ and a salt rejection rate of 99.7% (Fig. 5(a)). Comparing the PVA/PAN nanofiber composite membrane with the MD desalination membranes, their results are among the best of all reported MD membranes at 30–75 °C and NaCl concentrations of 1.5–20 wt% (Fig. 5(b)). This phenomenon is ascribed to the excellent fouling resistance inherent to hydrophilic PVA, while MD membranes are susceptible to fouling from elevated salt concentrations and organic contaminants. In contrast, a PVA/PSf membrane developed by the same group demonstrated a water flux of 124.8 ± 3.2 kg m^−2^ h^−1^ and a salt rejection rate of 99.9% when separating a 3.5 wt% NaCl solution at 70 °C. When subjected to a high-concentration NaCl solution with a mass fraction of 20%, the water flux of the PVA/PSf membrane was measured at 71.8 ± 3.2 kg m^−2^ h^−1^.^63^

Improving the water flux of PV desalination membranes is a critical challenge, often addressed by reducing the thickness of the selective layer or lowering the resistance of the support layer.^64^ Li *et al.* crosslinked PVA with 4-sulfonylphthalic acid (SPTA) to fabricate S-PVA/PAN composite membranes, which successfully reduced the PVA layer thickness from 76 μm to 800 nm, a decrease of 95 times.^65^ However, the water flux only increased by 5.3 times, suggesting that a thin and dry region near the downstream section of the PVA layer dominates the water transport characteristics. This finding underscores that merely reducing the thickness of the selective layer is insufficient to achieve extremely high water flux. Future research should focus on developing membrane materials that exhibit high water diffusion rates in the thin layers near the downstream side of the membrane.

**Fig. 6:**
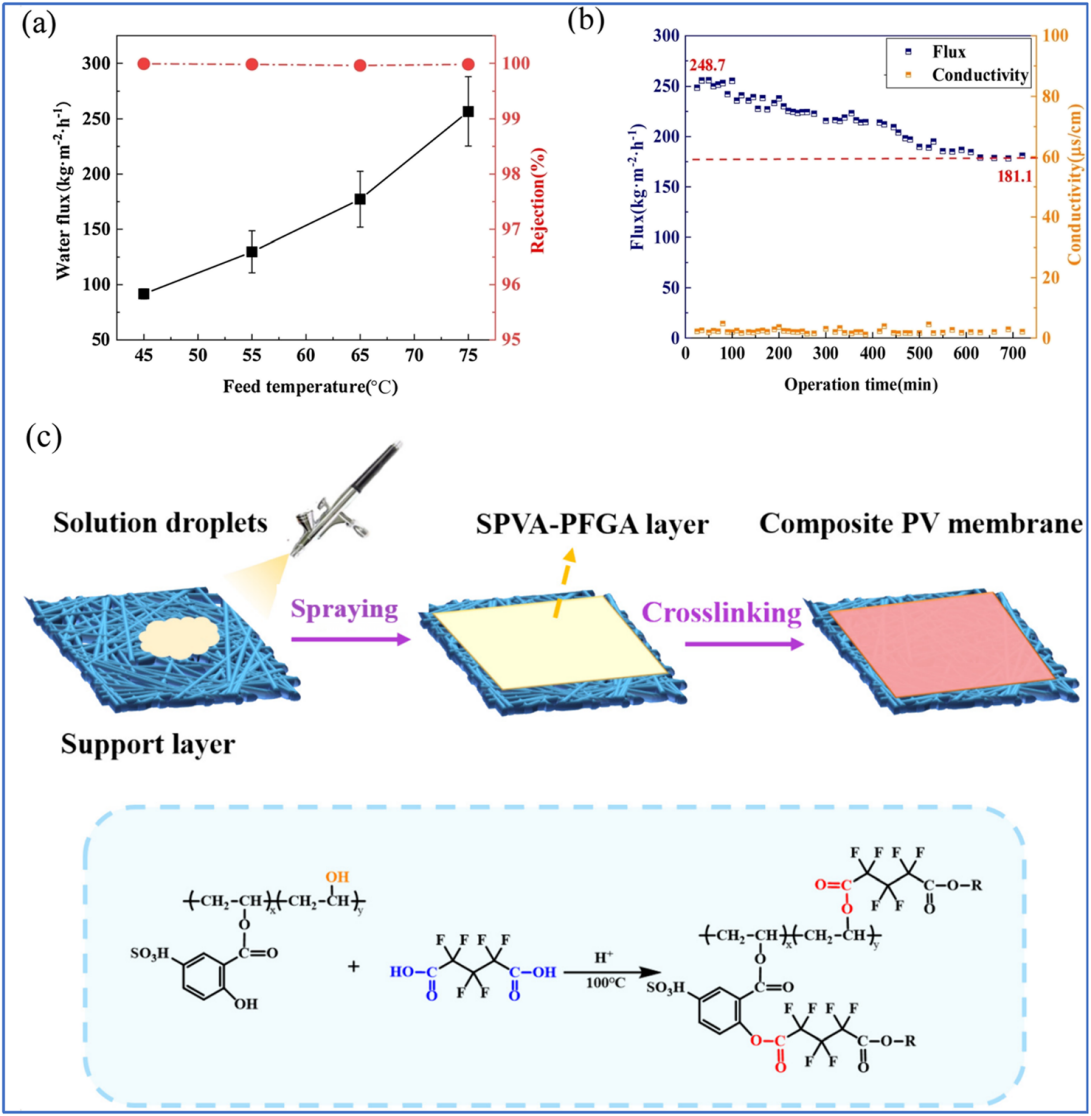
(a) Influence of feed temperature on water flux and salt rejection of the PVA-P(AA-AMPS) layer (3.5 wt% NaCl). (b) Influence of the porous plate on water flux and rejection of PV.^66^ (c) Illustration of the structure and preparation process of the composite membranes.^67^ Reproduced from ref. 66 and 67 with permission from Elsevier, copyright 2022 and 2024, respectively.

Furthermore, the solution coating method often employs a low-resistance support layer, which typically features larger surface pores and necessitates an increased skin layer thickness to prevent fracture. To address this issue, Cao *et al.* utilized P(AA-AMPS) crosslinked PVA to enhance the mechanical properties of the coating by spraying a PVA layer onto a polytetrafluoroethylene (PTFE) microfiltration membrane, and successfully fabricated a thin, defect-free S-PVA/PTFE composite membrane with a selection layer thickness of 1.1 μm.^66^ In a PV desalination experiment conducted at 75 °C on a 3.5 wt% NaCl aqueous solution, the membrane achieved a water flux of 256.6 ± 31.3 kg m^−2^ h^−1^ and a NaCl rejection rate exceeding 99.9% (Fig. 6(a and b)). Zhang *et al.* developed a novel composite membrane for PV desalination by modifying PVA with 5-sulfosalicylic acid (SSA) and perfluoroglutaric acid (PFGA) crosslinker and demonstrated significant improvements in both chlorine resistance and water permeability.^67^ The modified PVA (SPVA) exhibited enhanced hydrophilicity due to the introduction of sulfonic acid groups, which facilitated water transport across the membrane (Fig. 6(c)). Additionally, the use of PFGA as a crosslinking agent improved the membrane's chlorine resistance, allowing it to maintain high salt rejection even after prolonged exposure to sodium hypochlorite. Remarkably, it highlights the importance of chemical modifications in enhancing the performance and stability of PV membranes, providing valuable insights for the development of more robust desalination technologies.

Given the drawbacks of chemical crosslinking in PVA membrane preparation, such as its time-consuming nature and high reaction temperatures, photo-crosslinking appears to be a more sustainable and rapid method for future PVA membrane fabrication. Li *et al.* coated PVA-stilbazol quaternized (SBQ) onto electrospinning nanofiber membranes and subsequently photo-crosslinked the mixture for just 1 min to produce PVA-SBQ/nanocellulose-PAN composite membranes.^68^ In pervaporation experiments conducted at a feed temperature of 75 °C using a 3.5 wt% NaCl solution, the resulting water flux was 122.6 ± 10.8 kg m^−2^ h^−1^. Additionally, the membrane demonstrated excellent desalination performance with a 20 wt% NaCl solution, achieving a water flux of 79.9 ± 13.3 kg m^−2^ h^−1^. To further improve the desalination performance, Zhang *et al.* coated PVA-SBQ on a PTFE microfiltration substrate and integrated ZIF-8 inorganic fillers into the PVA layer, which not only reduced the water transport resistance and crystallinity of PVA but also improved the transport volume of the composite PV membrane.^69^ PVA-SBQ could be cross-linked under UV radiation within 1 min to afford stable cross-linked membranes. In pervaporation experiments conducted at a feed temperature of 80 °C using 3.5 wt% NaCl solution, the resulting water flux was 307.58 ± 15.09 kg m^−2^ h^−1^. Additionally, the membrane demonstrated excellent desalination performance with a 10 wt% NaCl solution, achieving a water flux of 129.95 to 152.68 kg m^−2^ h^−1^. Notably, the fabrication of such a composite PV desalination membrane is easily achievable due to the ease of performing UV light-induced cross-linking of the commercial PTFE substrate and ZIF-8 nanofillers. Zachariah *et al.* developed a novel approach by incorporating the biomimetic structure and chemical composition of the lotus leaf surface onto a hydrophilic polymer-based membrane, which leads to deprotonation of the PVA membrane surface, an increase in the membrane surface area, and a significant enhancement in its hydrophilicity.^70^ Consequently, the water permeation flux of the modified PVA membrane was found to be 2.8 times higher than that of the original PVA membrane. The authors suggested that this method primarily targets the surface structure of the membrane and can be effectively combined with other membrane modification techniques. This approach holds promising applications in the preparation of composite membranes, reduction of selective membrane thickness, and the establishment of water channels within the membrane.

In addition, the incorporation of high-performance fillers into PVA to fabricate composite membranes represents an effective strategy for enhancing the properties of PVA-based membranes. An *et al.* developed a novel thin-film nanocomposite (TFN) membrane incorporating amino-embedded carbon quantum dots (ACQDs) for PV desalination and demonstrated that the incorporation of ACQDs significantly enhanced the water flux by 44% while maintaining high salt rejection of 99.9%.^71^ The ACQDs not only improved the hydrophilicity of the membrane but also provided excellent chlorine resistance and fouling resistance, making the membrane more suitable for long-term desalination applications. This work underscores the importance of integrating nanomaterials into PV membranes to enhance their performance and stability, a strategy that is increasingly being explored in the field of desalination. Darmawan *et al.* developed a novel design of a composite membrane composed of GO, PVA, and a Zn(ii) cross-linker on a macroporous nylon substrate.^72^ This research highlighted the importance of optimizing membrane composition to achieve high water flux and salt rejection rates, which are critical parameters for efficient desalination. The use of GO—known for its hydrophilic properties and high surface area—in conjunction with PVA as a hydrophilic polymer and Zn(ii) ions as cross-linkers, demonstrates a strategic approach to enhance both the stability and permeability of the membrane.

### 3.2. Cellulose and its derivatives

Among the various membrane materials explored for PV, cellulose and its derivatives polymers have garnered significant attention due to their unique properties, such as easy accessibility, excellent hydrophilicity, better water permeability, good biodegradable and biocompatible nature and great toughness.^73^ These polymers, including cellulose (Fig. 3), and its chemically modified derivatives, such as cellulose diacetate (CDA), cellulose triacetate (CTA), cellulose acetate (CA), nanocellulose (*e.g.*, cellulose nanocrystals (CNCs), bacterial cellulose (BNC) and cellulose nanofibrils (CNFs)) and carboxymethyl cellulose (CMC), *etc.*, have been extensively studied for their potential in enhancing the efficiency of pervaporation processes.^74–77^ However, several inherent disadvantages of cellulose and its derivatives limit their widespread application in PV desalination processes, *e.g.*, mechanical weakness, low thermal stability and fouling/chlorine susceptibility. These disadvantages have prompted researchers to explore various strategies to overcome these limitations, such as blending cellulose with other polymers (*e.g.*, polyvinyl chloride (PVC) and polyethylene glycol (PEG)), incorporating nanoparticles (such as GO, nano-clays, SiO_2_, Al_2_O_3_, TiO_2_, CNCs), and applying surface modification techniques (for example, surface plasma treatment and chemical modification) to enhance the mechanical strength, chemical stability, and fouling resistance of cellulose-based membranes, thereby improving their suitability for pervaporation desalination processes.^78–80^

Combining the characteristics of different polymers to enhance membrane performance is an effective approach to improve the water flux and desalination efficiency of PV membranes. Abdallah *et al.* blended CA membranes with PVC to enhance the fouling resistance of the CA membranes, and reported that the fouling rejection rate, salt rejection rate, and permeation flux were 96.0–99.2%, 93.3%, and 13 L m^−2^ h^−1^, respectively.^81^ In addition, the blended membrane exhibited outstanding mechanical properties and the results of the stress–strain test showed a strength of 12.6 N cm^−2^ and an elongation of 25 mm. Ahmad *et al.* demonstrated that the addition of hydrophilic PEG into CA membranes significantly enhanced their permeation performance and fouling resistance.^82^ PEG improved the hydrophilicity of the membrane, leading to an increase in water flux from 0.35 to 2.46 L m^−2^ h^−1^, along with an 11.41% relative increase in salt rejection. This improvement in performance can be attributed to the increased surface hydrophilicity and the creation of additional pathways for water transport within the membrane matrix. Although the water flux of cellulose-based membranes could be modified by blending other polymers, the mechanical properties and water permeability should be further improved. Ongoing research is focused on the development of advanced blending techniques, and the use of compatible and functional polymer combinations.

**Fig. 7:**
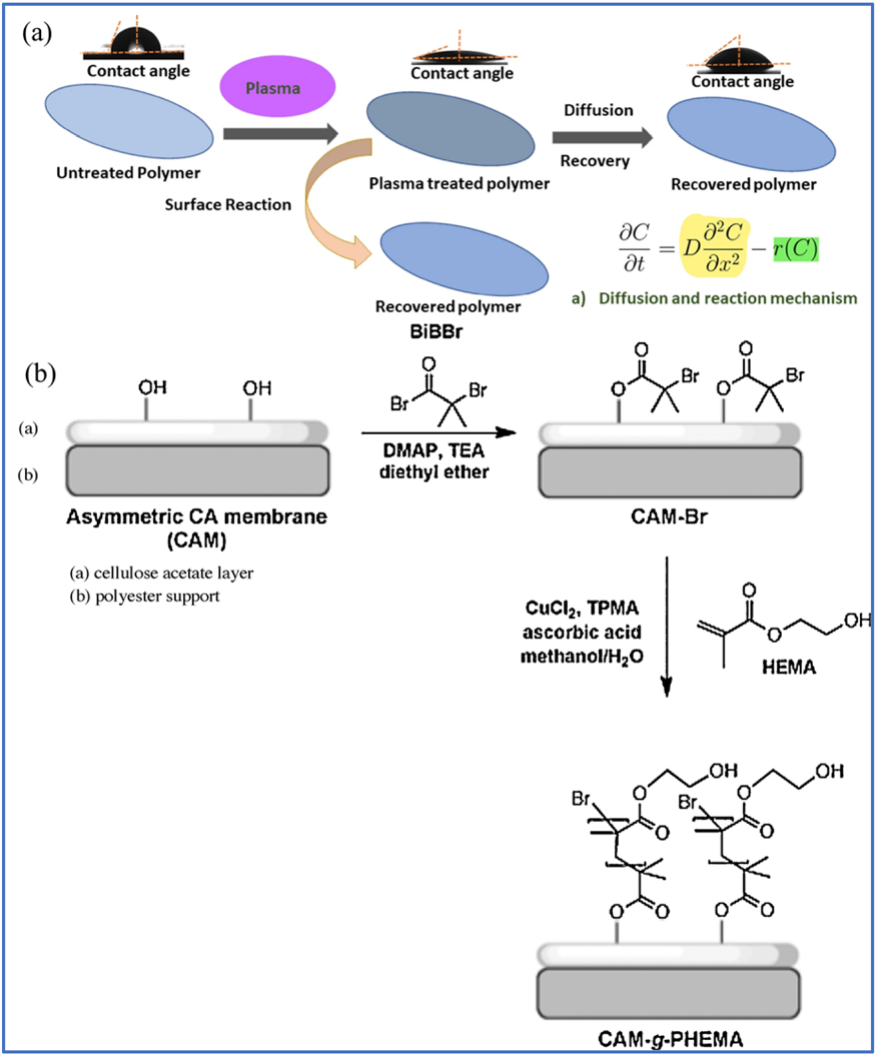
(a) Diffusion and reaction mechanism of plasma treated polymer membrane.^88^ (b) Illustration of the reaction process for surface modification of CAM.^91^ Reproduced from ref. 88 and 91 with permission from *Nature* and Elsevier, copyright 2024 and 2024, respectively.

Surface plasma treatment is a versatile and widely used technique in membrane applications, primarily aimed at modifying the surface properties of membranes to enhance their performance in various processes.^83–85^ This treatment involves exposing the membrane surface to a plasma environment, which can introduce reactive functional groups, alter surface chemistry, and modify surface topography. Bhat *et al.* found that the surface hydrophilicity of CTA membranes could be improved after ammonia plasma treatment, resulting in an improvement in water flux.^86^ Shinbo *et al.* investigated the water flux and selectivity of oxygen plasma treated CA membrane.^87^ The results showed that the water flux slightly decreased while the selectivity greatly increased, which is due to the introduction of oxygen-containing anionic groups on the surface of CA after oxygen plasma treatment. Very recently, Hazarika *et al.* provided valuable insights into the role of N_2_ and Ar plasma-treated membranes in enhancing the performance of desalination systems (Fig. 7(a)).^88^ The findings reveal that plasma treatment significantly improves the hydrophilicity and permeate flux of these membranes, which are crucial factors for efficient desalination. Specifically, Ar plasma treatment resulted in a higher permeate flux and rejection rate compared with N_2_ plasma, attributed to the increased pore size and surface roughness of the membrane.

Chemical modification is another widely used method to enhance the performance of membrane surfaces, particularly in applications where fouling resistance and hydrophilicity are critical, which involves introducing functional groups or coatings onto the membrane surface through chemical reactions.^89^ Naim *et al.* used the phase inversion method, dissolving CA in a mixture of acetone, dimethylformamide, dimethyl phthalate, and glycerol, and then subjected it to surface treatment to obtain a CA membrane with a super-hydrophilic surface.^90^ The prepared CA membrane has a water permeability of 5.97 L m^−2^ h^−1^ and a desalination efficiency of 99.7%. Worthley *et al.* reported surface modification of CA membranes by atom transfer radical polymerization (ATRP) to obtain CA-grafted hydrophilic 2-hydroxylethyl methacrylate membranes, which exhibited 24% higher resistance to microbial biofouling in seawater while maintaining salt rejection and water flux properties (Fig. 7(b)).^91^ However, surface plasma treatment suffers from the drawbacks of temporary hydrophilicity, possible material degradation, complexity and cost, and limited industrial scalability. Meanwhile, the chemical modification method withstands the disadvantages of environment concerns, complexity and cost, and limited scalability.

The incorporation of nanofillers into cellulose-based membranes can significantly enhance water permeability by providing additional pathways within the polymer matrix. Moreover, the mechanical properties of nanocomposite membranes are generally superior to those of their pristine counterparts, especially given their typically thin structure. A nano composite CTA/SiO_2_ membrane was prepared by blending CTA with low cost, toxic and highly hydrophilic nano-SiO_2_ to enhance the mechanical properties of CTA.^92^ The researchers systematically investigated the effects of varying SiO_2_ loading (from 1 to 4 wt%) on the membrane's morphology, mechanical properties, and desalination performance. Their findings revealed that incorporating 4 wt% SiO_2_ into the CTA membrane significantly increased the water flux by a factor of 2.5 from 2.2 to 6.1 kg m^−2^ h^−1^ for a 30 g per L NaCl feed solution at 70 °C, without compromising the salt rejection (remained above 99%), which was attributed to the enhanced hydrophilicity and additional permeation pathways provided by the SiO_2_ nanoparticles. Furthermore, the same group presented a significant advancement of CTA-based membrane's desalination performance by developing CTA/Al_2_O_3_ nanocomposite membranes for PV desalination.^93^ Their findings demonstrated that incorporating 2 wt% Al_2_O_3_ into the CTA membrane significantly increased the water flux by 204% from 2.2 to 6.7 kg m^−2^ h^−1^ for a 30 g per L NaCl feed solution at 70 °C compared with pristine CTA, while maintaining a salt rejection of over 99.8%. This enhancement is attributed to the improved hydrophilicity and additional permeation pathways provided by the Al_2_O_3_ nanoparticles. In addition, the study shows that the nanocomposite membrane only exhibits a 25% flux reduction when tested with a higher NaCl concentration of 90 g L^−1^, indicating its robustness and potential for treating hypersaline solutions.

**Fig. 8:**
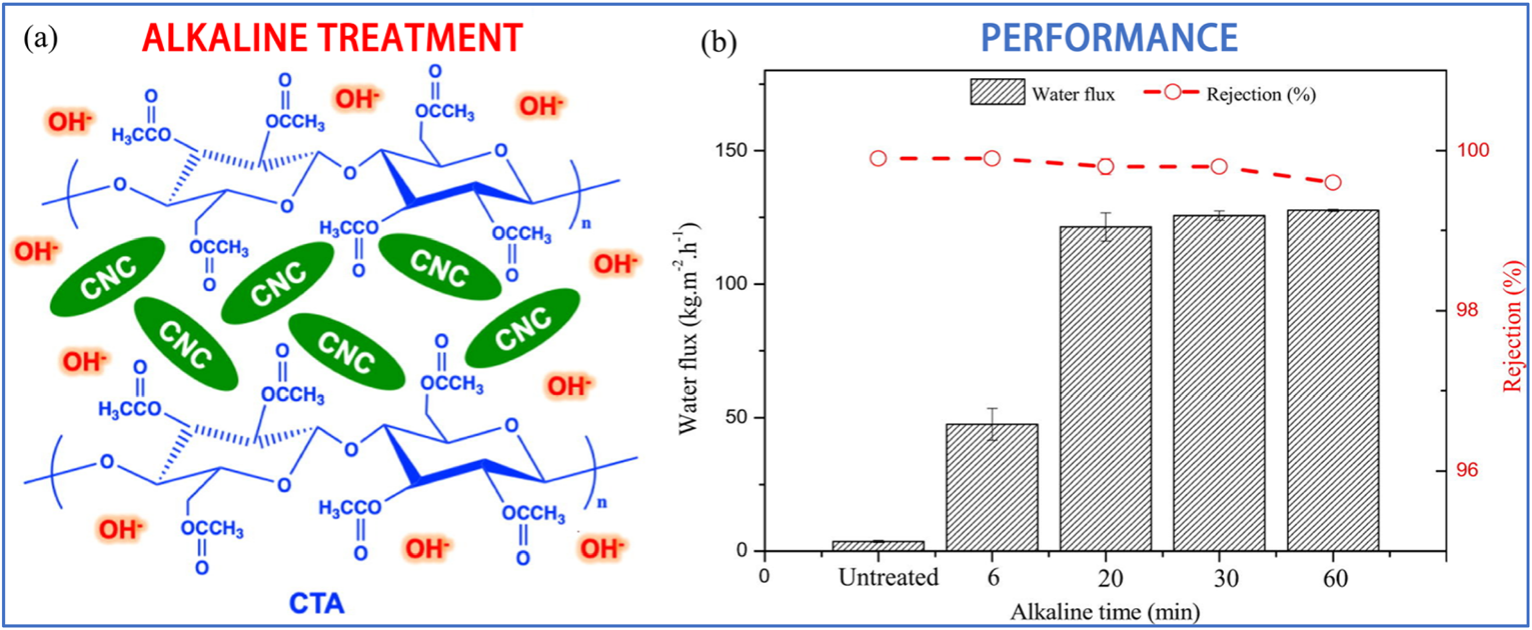
(a) Illustration of the alkaline treatment of CTA/CNC composite membrane. (b) Dependance of water flux and salt rejection of CTA/CNC composite PV membrane on alkaline treatment time (3.5 wt% NaCl).^95^ Reproduced from ref. 95 with permission from Elsevier, copyright 2021.

CNCs are a rapidly developing class of nanomaterials endowed with excellent hydrophilicity characterized by the presence of hydroxyl, sulfonic acid, and carboxylic acid functional groups on their surfaces, robust mechanical properties, and cost-effective features. Prihatiningtyas *et al.* incorporated 3 wt% CNCs into the CTA casting solution to create CTA/CNC blend membranes, which not only altered the membrane's microstructure but also enhanced its mechanical strength.^94^ The water flux increased threefold from 2.16 kg m^−2^ h^−1^ to 5.76 kg m^−2^ h^−1^. By decreasing the scraper height from 200 μm to 100 μm, the membrane thickness was further reduced, resulting in a flux of 11.68 kg m^−2^ h^−1^ and a NaCl rejection rate of 99.9%. Although the water flux of such composite membranes was improved, it was still poor in comparison with other pervaporation desalination membranes. With this in mind, they further treated the CTA/CNC composite membranes under alkaline conditions (Fig. 8(a)).^95^ Interestingly, the hydroxyl group content of the CTA/CNC nanocomposite membranes could be well-controlled with time, and the water contact angle decreased from 65.6° to 24.7°. The pervaporation test was conducted on a 9 wt% NaCl aqueous solution at 70 °C, and the water flux rose from 3.6 kg m^−2^ h^−1^ to 107.5 kg m^−2^ h^−1^, while the salt rejection rate remained at 99.9% (Fig. 8(b)). When the NaCl mass fraction was increased to 20 wt%, the water flux was 58.5 kg m^−2^ h^−1^, and a salt rejection rate of over 99.8% was maintained. This treatment improves the hydrophilicity of the membrane by increasing the density of hydrophilic functional groups, thereby facilitating better water transport through the membranes without comprising other properties.

BNCs are natural polymers synthesized by microorganisms such as those from the genus *Acetobacter*, possessing a high degree of crystallinity (84–95%) endowing exceptional mechanical strength (with a Young's modulus of 200–300 MPa) and stability, with a superhydrophilic three-dimensional nanofiber network (providing a high specific surface area and a dense porous structure) exhibiting high water retention capacity due to the abundance of hydroxyl groups on their surface.^96–98^ Consequently, the range of applications for BNC continues to broaden, encompassing fields such as bioprocessing, biomedical and pharmaceutical sectors, the food industry, wastewater treatment, and numerous other areas. However, the exploration of their potential in fabricating membranes tailored for PV desalination remains relatively limited, which may be due to challenges such as high production costs (low efficiency of traditional fermentation processes, and the need to develop low-cost substrates) and insufficient long-term stability, where BNC may undergo swelling or structural collapse in high-salt or high-temperature environments, necessitating the enhancement of durability through cross-linking or the incorporation of hydrophobic materials.^99^

### 3.3. PAN

PAN is a semicrystalline organic polymer characterized by its repetitive nitrile (–CN) moieties linked to polyethylene backbones (Fig. 3) that exhibits both hydrophilic properties and the capacity to engage in hydrogen bonding due to the lone electron pair on nitrogen atoms.^100,101^ This feature imparts a significant dipole moment across nitrogen and carbon atoms, facilitating robust intermolecular attractions that are pivotal for membrane fabrication, surface functionalization, and grafting modifications. The inherent strength and resistance of PAN chains to various organic solvents—stemming from their intermolecular interactions—make them ideal for constructing PV and organic solvent filtration membranes.^102,103^ The limited solubility of PAN in conventional organic solvents and the exceptional mechanical attributes of its fibers (attributed to the polymer chain interactions) have positioned PAN as a prime material for nanofiber-based membrane production.^104^ Since 1974, PAN-based membranes have been extensively utilized in the development of separation membranes, with applications expanding across diverse processes such as water treatment, organic solvent purification, and fuel cell technologies, owing to their commendable thermal and chemical stability, as well as solvent resistance.^105–107^ Despite its advantageous properties, PAN exhibits suboptimal biocompatibility and hydrophilicity, which can result in cell adhesion and protein adsorption on the membrane surface, potentially inducing biofouling, especially in applications involving blood separation. To address these challenges, recent research has focused on developing physicochemical modification strategies for PAN-based PV membranes, aiming to improve the biocompatibility, hydrophilicity, and antifouling characteristics of PAN, thereby enhancing its performance in PV desalination applications.

**Fig. 9:**
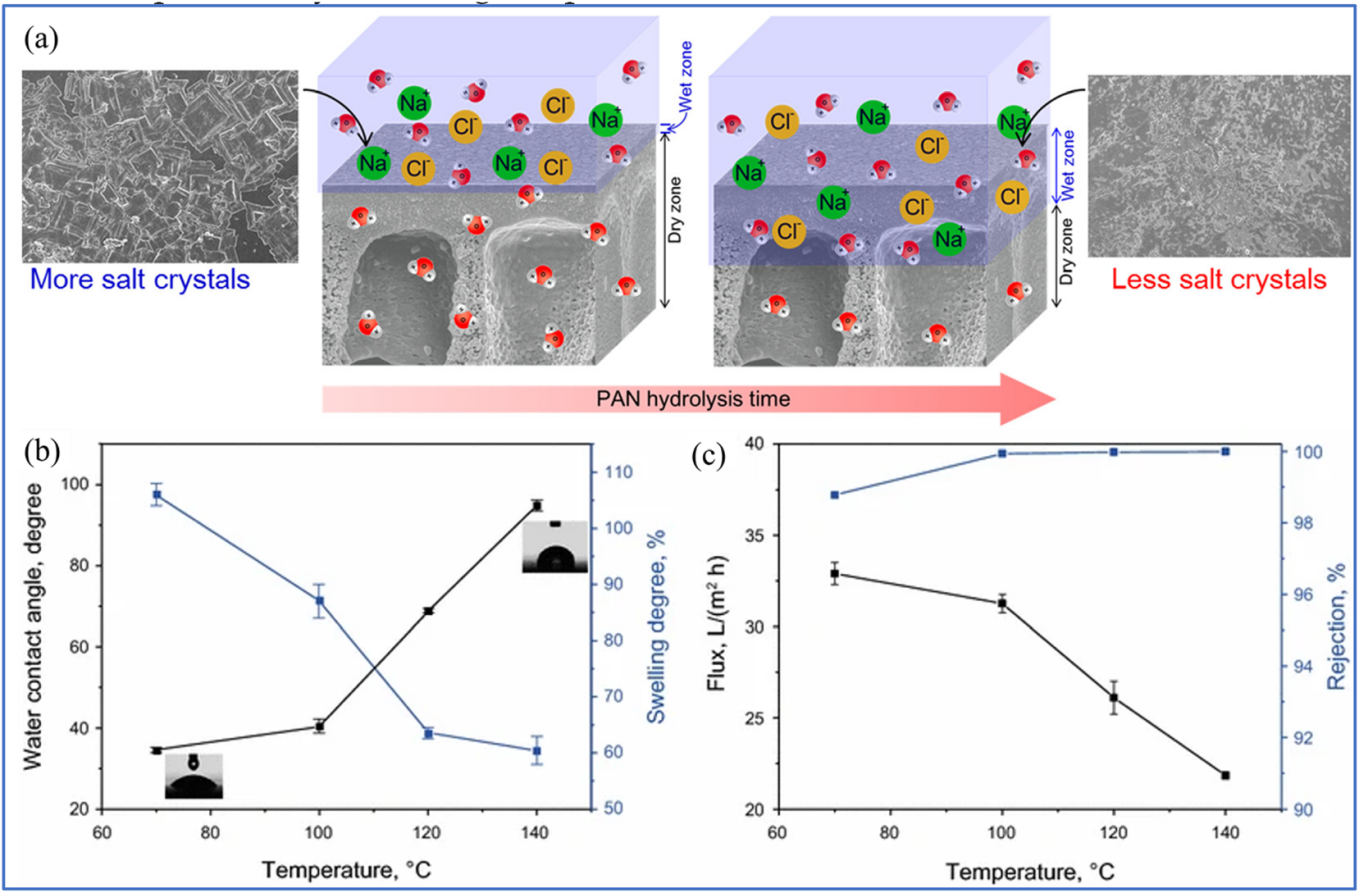
(a) The proposed mechanism for PV desalination using hydrolyzed PAN porous membranes.^108^ (b) The water contact angles and swelling properties. (c) The water flux and salt rejection (using the 35 000 ppm NaCl solution as the feed at 70 °C) of M-d composite membranes crosslinked at different temperatures for 2 h.^109^ Reproduced from ref. 108 and 109 with permission from Elsevier, copyright 2019 and 2018.

Austria *et al.* fabricated PAN membranes using the diffusion-induced phase separation (DIPS) method and further modified these membranes through hydrolysis to enhance their hydrophilicity and tune their microstructure morphology.^108^ This method typically results in a highly porous and open substructure, featuring a thin upper layer and a porous sublayer with macrovoids. They found that an extended hydrolysis period of the PAN membrane leads to a brief free volume and the breakdown of intermolecular hydrogen bonds in water creates a thick wet zone and a narrower dry zone (Fig. 9(a)). Consequently, salt crystallization on the surface is reduced, maintaining a high salt rejection rate of >99.9% over 72 h of operation. At shorter hydrolysis times, the membrane shows a low degree of swelling, resulting in a moderately thin wet zone and a thicker dry zone. These findings suggest that membranes lacking top layers could also be effective in PV desalination, potentially reducing the production costs of such membranes.

Zhang *et al.* engineered PVA/PAN composite membranes, utilizing pyromellitic dianhydride (PMDA) as a cross-linking agent for the desalination of wastewater.^109^ To bolster the mechanical integrity of the PVA membrane, a PAN ultrafiltration (UF) support was employed. An increase in the concentration of PMDA within the PVA dense layer correlated with enhanced water flux and NaCl rejection rates, which was likely attributed to the facilitated transport properties of PMDA molecules, mediated by carboxyl (–COOH) groups. To optimize this process, PMDA molecules were subjected to hydrolysis using sulfuric acid. Notably, the PVA/PAN membrane cross-linked with 20 wt% PMDA that was cured at 100 °C for 2 h demonstrated a water flux of 32.26 L m^−2^ h^−1^ at 70 °C and achieved a NaCl rejection of 99.98% when treating a feed solution with 35 000 ppm of NaCl (Fig. 9(b and c)).

Han *et al.* reported an enhancement in the tensile strength of asymmetric PAN membranes through the incorporation of kaolin, which also improved the membrane's hydrophilicity and led to the formation of a uniform, finger-like pore structure, thereby increasing mass transfer efficiency.^110^ Under alkaline conditions, the functional groups and pore structure of the membrane were further modified through hydrolysis, converting the surface CN groups to CONH_2_ and subsequently to COOH. The formation of COOH groups resulted in the compaction of the top selective layer, which ensured high salt rejection efficiency. In PV experiments, PAN membranes blended with 2% kaolin demonstrated a water flux of 82 kg m^−2^ h^−1^ in a 3.5 wt% NaCl solution and 59 kg m^−2^ h^−1^ in a 10 wt% NaCl solution, with a NaCl rejection rate of 99.99%. Long-term operational tests revealed that the HPAN-2 membrane exhibited stable PV desalination performance in a 10 wt% NaCl solution at 30 °C.

**Fig. 10:**
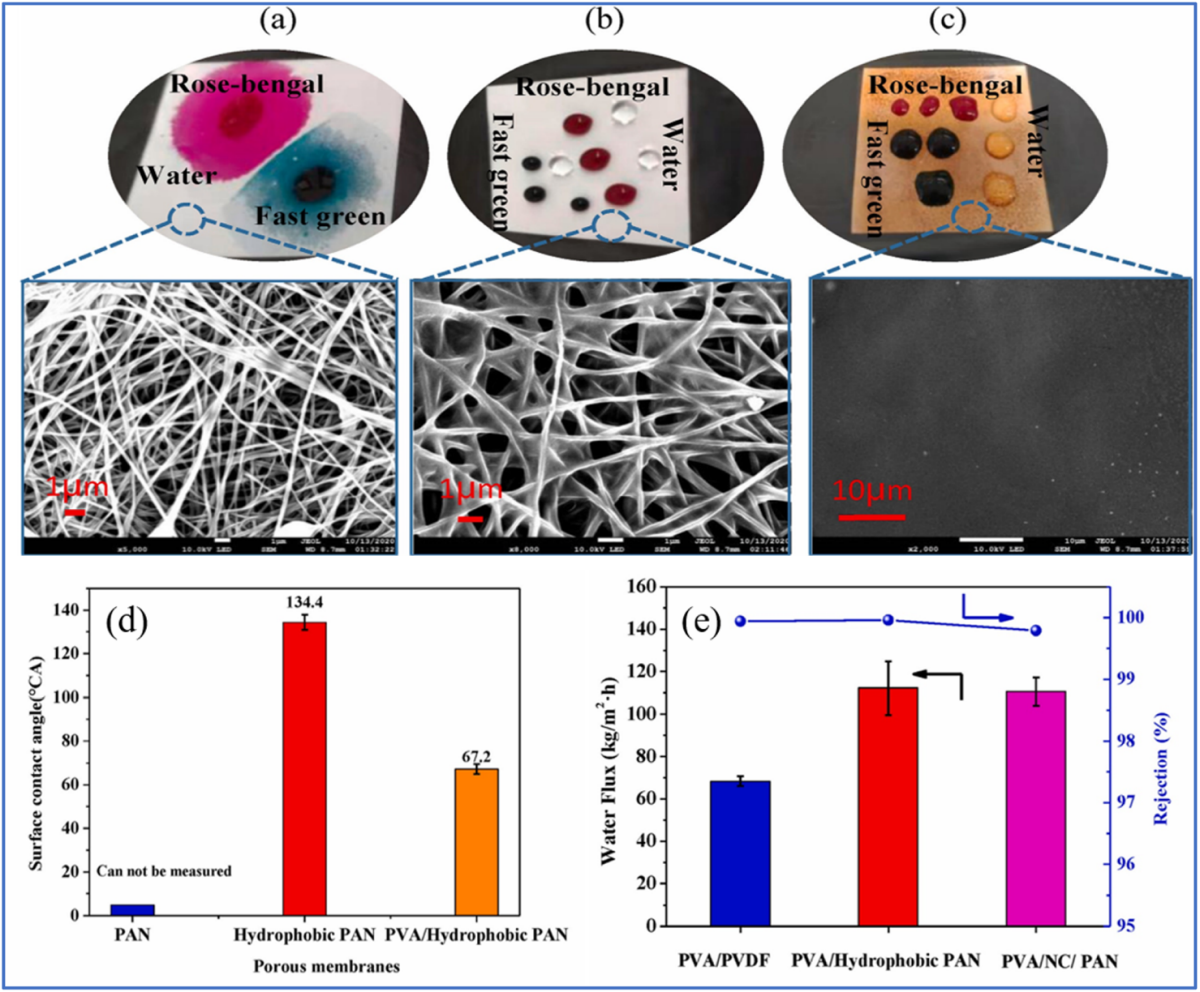
SEM images of dyes and water droplets on the surface of membranes: (a) pristine PAN, (b) hydrophobic PAN, (c) PVA/hydrophobic PAN, (d) water contact angles of different membranes and (e) water flux and salt rejection of different composite PV membranes (3.5 wt% NaCl).^111^ Reproduced from ref. 111 with permission from Elsevier, copyright 2021.

Li *et al.* devised two straightforward yet effective methodologies to improve the PV desalination performance of PAN membranes.^111^ The first involved altering the surface characteristics of a PAN nanofiber membrane through the application of a hydrophobic, waterproof coating. Subsequently, a substantial layer of polyvinyl alcohol (PVA) was deposited onto this hydrophobically altered PAN nanofiber support. The second approach entailed the creation of an intermediate layer between the PAN substrate and the PVA overlay to prevent PVA penetration. The majority of PVA molecules were repelled by a CNF barrier layer, leading to the formation of a thin, defect-free PVA upper layer (700 nm) applied *via* spray coating onto the NC-PAN substrate. Ultimately, a thin-film composite (TFC) pervaporation membrane was successfully fabricated. Fig. 10(a–c) illustrate the dispersion of dye and water droplets on the surfaces of untreated PAN, hydrophobic PAN, and PVA-coated hydrophobic PAN nanofiber membranes. The unmodified PAN nanofibrous membrane exhibits inherent hydrophilicity. Following the application of fluorinated paint, a water contact angle of 134.4° was achieved (Fig. 10(d)), indicating a transition to a hydrophobic surface. A uniformly distributed PVA layer was subsequently formed atop the hydrophobically modified PAN membrane using spray-coating technology. The water contact angle of the TFC membrane was reduced to 67.2° through cross-linking of the PVA layer. The TFC pervaporation membrane demonstrated a water flux of 238.7 kg m^−2^ h^−1^ at 80 °C with 99.8% rejection for a 3.5 wt% NaCl solution (Fig. 10(e)). A comparison between the fabricated PAN support and a polyvinylidene fluoride (PVDF) support highlighted the superior performance of the novel PAN-based support.^112^

### 3.4. PA

PV membranes of PA endowing excellent performance and durability (*e.g.*, high thermal and chemical stability) are typically formed through IP, which involves the reaction of two monomers (one in the aqueous phase and the other in the organic phase) to form a TFC structure; the method allows for the creation of membranes with high selectivity and permeability, making them suitable for removing salts and other impurities from water.^113–116^ One of the primary advantages of polyamide membranes is their ability to achieve high salt rejection rates, often exceeding 98%, which is crucial for seawater desalination applications. Generally, the structure of PA membranes includes a thin PA layer on top (separation layer) of a porous support layer, typically made of PVA or CA, which enhances both the mechanical strength and the separation efficiency of the membranes.^117,118^ Commonly, PA is synthesized by the IP of organic-phase reactive monomer TMC with aqueous-phase reactive monomer (*m*-phenylenediamine, MPD) (Fig. 3, PA-1) or PIP (Fig. 3, PA-2) on the surface of the support layer.

**Fig. 11:**
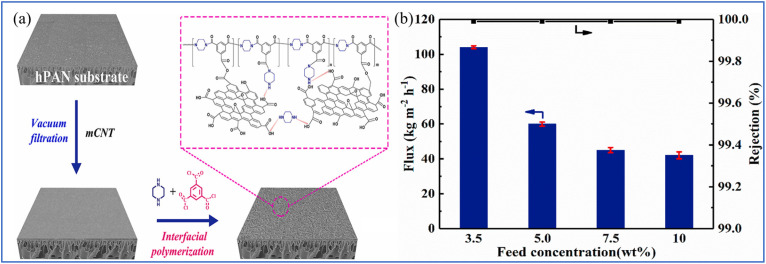
(a) Illustration of the preparation of PA-mCNT on the hPAN ultrafiltration membrane. (b) Dependance of water flux and salt rejection on feed concentration.^120^ Reproduced from ref. 120 with permission from Elsevier, copyright 2022.

Recent advancements have focused on improving the hydrophilicity of PA membranes to increase water flux while maintaining salt rejection. Hydrophilic additives, such as GO, CNTs, amino acids and other nanomaterials, and polymers (*e.g.*, PVA) have been incorporated into the membrane formulation either as a monolayer or multilayer structure, significantly improving the membrane's hydrophilicity and water permeability properties.^119^ For instance, the incorporation of GO into polyamide membranes improves both the flux and antifouling properties of PA membranes by increasing their hydrophilicity. The addition of GO not only enhances the membrane's hydrophilicity but also provides additional mechanical strength and chemical stability, making it a promising additive for desalination applications. Zhang *et al.* synthesized multi-walled CNTs (MWCNTs) in a core–shell configuration and employed modified carbon nanotubes (mCNTs) as interstitial scaffolds between the PA layer and the PAN ultrafiltration membrane (Fig. 11(a)).^120^ The mCNTs were applied to the PAN ultrafiltration membrane to facilitate IP reactions, resulting in a novel PA composite membrane (PA-mCNT). Permeation experiments conducted at 70 °C using solutions with mass fractions of 3.5 wt% and 10 wt% NaCl yielded water fluxes of 104 and 40.8 kg m^−2^ h^−1^, respectively, while maintaining salt rejection rates of 99.99% (Fig. 11(b)).

Although the above additives could improve the water flux and salt rejection performance of PA composite membranes, they pose disadvantages in the multistep manufacturing process, with high cost and environment issues. In another innovative strategy, amino acids (such as glycine and l-lysine) co-monomers in IP modifications have shown promising results in enhancing both desalination performance and the removal of specific contaminants like arsenate for semi-aromatic PA-based PV membranes.^121^ The versatile modification resulted in a significant increase in permeation on flux without compromising salt rejection rates, which remained above 99.8%. In addition, the study also underscored the substantial influence of operating temperature on membrane performance, with permeation flux increasing substantially as the temperature rose from 40 °C to 70 °C. These findings are particularly relevant as they demonstrate a cost-effective and environmentally friendly method for improving the performance of PV membranes.

It should be noted that PA membranes are susceptible to chlorination reactions under alkaline conditions, with their aromatic rings particularly susceptible to chlorine attack.^122,123^ Treatment with diluted hypochlorite can enhance the hydrophilicity and negative charge of the membrane, facilitating cation aggregation. Halakoo *et al.* surface-treated PA membranes and subsequently deposited alternating layers of positively charged polyethyleneimine (PEI) and negatively charged GO to form a PEI/GO layer-by-layer self-assembly (LbL) membrane.^124^ This chlorination treatment improved membrane permeability, and the addition of PEI and GO to the chlorinated PA membranes increased its salt rejection rate. The pure water flux of the PEI/GO LbL membrane was double that of the original membrane and the water flux reached 8.4 kg m^−2^ h^−1^ with a salt rejection rate of 99.9% at 65 °C using a 20 wt% NaCl mass fraction aqueous solution. Zhang *et al.* developed a novel PA-based TFC membrane using a new N-rich amine monomer (3,5-diamino-1,2,4-triazole, DAT) in the IP process to enhance the chlorine resistance and PV desalination performance.^125^ The optimal membrane exhibits an exceptionally high pure water permeability of 95.1 L m^−2^ h^−1^ bar^−1^ and outstanding selectivity for separating Congo red and NaCl, achieving rejection rates of 99.1% and 5.8%, respectively. The unique molecular structure of DAT contributes to the formation of a less compact polyamide layer, resulting in improved permeability and selectivity for dye/salt separation.

### 3.5. Sulfonated block polymers

Sulfonated block polymers integrate the attributes of ion-crosslinked polymers and thermoplastic elastomers and the sulfonic acid moieties within the polymer backbone enhance the diffusion of water and other polar molecules.^126,127^ Sulfonated aromatic polymers (SAPs) possess unique nanochannel structures, exhibit selective water permeability, and demonstrate excellent physical and chemical stability, rendering them promising candidates for seawater desalination membranes. Notably, sulfonated polystyrene/styrene–butadiene–styrene (S-SEBS) is a sulfonated aromatic polymer characterized by a highly hydrophobic main chain and abundant hydrophilic groups. Wang *et al.* synthesized a sulfonating agent (acetyl-sulfate) from concentrated sulfuric acid and acetic anhydride, and grafted sulfonic acid groups onto SEBS triblock copolymers to produce a sulfonated aromatic polymer, as depicted in Fig. 6, and the performance of S-SEBS membranes with varying degrees of sulfonation in pervaporation desalination was investigated.^128^ The degree of sulfonation increased from 0 to 54.1%, leading to a corresponding rise in the water absorption rate from 0 to 188.9%, while the membrane contact angle decreased from 100° to 23°. The rejection rate of various salts exceeded 98.4%, with a flux of 22.87 kg m^−2^ h^−1^.

**Fig. 12:**
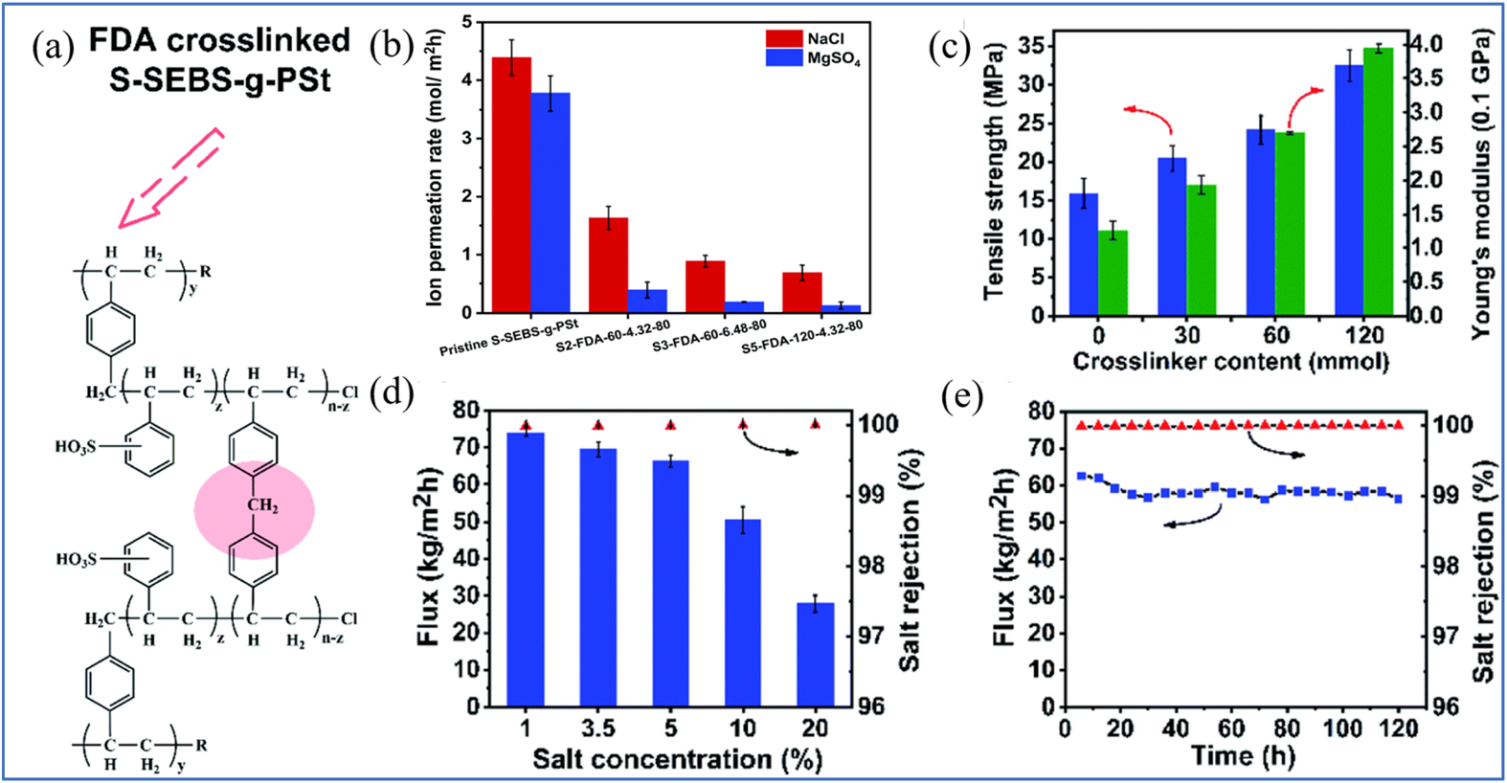
(a) Chemical structure of FDA-crosslinked S-SEBS-*g*-PSt membrane. (b) Ion permeation rate of pristine and FDA-crosslinked S-SEBS-*g*-PSt membranes. (c) Tensile strength and young's modulus of S-SEBS-*g*-PSt membrane. (d) and (e) Effect of the feed concentration and long term performance of FDA-crosslinked S-SEBS-*g*-PSt membranes (65 °C).^129^ Reproduced from ref. 129 with permission from The Royal Society of Chemistry, copyright 2022.

Xie *et al.* grafted polystyrene onto the side chain of S-SEBS and crosslinked it using formaldehyde (FDA) as a crosslinking agent to form methylene bridges (Fig. 12(a)).^129^ The combination of polystyrene grafting and FDA crosslinking resulted in a more stable microstructure, with a significant reduction in ion permeation rates compared with the original membranes, which has a positive effect on salt rejection performance (Fig. 8(b)). Remarkably, the tensile strength and Young's modulus of the crosslinked structure of the membranes also increased to 32.5 MPa and 3.95 GPa, respectively, which is crucial for the anti-swelling performance and the long-term stability of the membranes (Fig. 12(c)). When treating a 5 wt% NaCl solution at 65 °C, the membrane exhibited a water flux of 76.8 kg m^−2^ h^−1^ with a salt rejection rate of 99.98% (Fig. 12(d)). Additionally, the water flux was sustained at an average of 58 kg m^−2^ h^−1^ upon daily rinsing, with salt rejection efficiency consistently reaching 99.9% (Fig. 12(e)). The above results suggest that the introduction of benzene rings and crosslinking of sulfonated polymers could be a promising application potential for desalination by PV membranes.

Although highly sulfonated aromatic polymers (SAPs) possess active functional groups that enhance water permeation, their membranes can excessively expand and suffer from deteriorated mechanical properties in high-concentration aqueous environments. Yan *et al.* employed a hydroxyl-terminated hyperbranched polyester (H302) as a crosslinking agent to modify the S-SEBS copolymer membrane.^130^ The crosslinking process is illustrated in Fig. 7. Experimental results revealed that the unique topological structure of H302 facilitates the transport of solvent molecules by disrupting the dense entanglement of S-SEBS chains, thereby increasing the material's free volume and enhancing the diffusion rate and permeation flux. Following thermal crosslinking, the mechanical strength of the S-SEBS membrane significantly improved, with the tensile strength increasing by 140% to 200% and the swelling rate decreasing by 45% to 70%, while maintaining a higher water flux. When used to treat 5% high-salinity water at 65 °C, the S-SEBS membrane containing 15% H302 achieved a water flux of 9.3 kg m^−2^ h^−1^ and a salt rejection rate of 99.9%.

Sulfonated pentablock ternary copolymer (Nexar™) is a commercially available sulfonated pentablock copolymer and the structure is similar to S-SEBS. The copolymerization of *tert*-butyl styrene, ethylene, and propylene at both ends ensures the mechanical stability and chain flexibility of the membrane.^126^ Thomas *et al.* compared Nexar™ with commercial MD and PV membranes and found that Nexar™ exhibits superior desalination and permeation performance compared with commercial PV membranes, and comparable performance to commercial MD membranes.^131^ In their study, the best-performing membrane achieved a permeability of 1355 ± 290 kg m^−2^ h^−1^ MPa^−1^, a flux of 7.1 ± 1.5 kg mm m^−2^ h^−1^, and a salt rejection rate of 99.5%. Table 1 summarizes the PV desalination performance of some polymer-based membranes.

**Table 1:** Summary of the PV desalination performance of some polymer-based membranes

Membranes		Salt concentrations/wt%	Temperatures/°C	Water fluxes/kg m^−2^ h^−1^	Salt rejection rate/%	Reference
PVA	PVA/PAN-P(AASS)	1.5	75	234.9 ± 8.1	99.7	62
	PVA/PSf	3.5	70	124.8 ± 3.2	99.9	63
	S-PVA/PAN	3.5	65	46.3	99.5	65
	S-PVA/PTFE	3.5	75	256.6 ± 31.3	99.9	66
	SPVA	3.5	70	120.38 ± 1.72	99.93	67
	PVA-SBQ/nanocellulose-PAN	3.5	75	122.6 ± 10.8	99.9	68
	PVA-SBQ/ZIF-8	3.5	80	307.58 ± 15.09	99.9	69
	BL-PVA	3.5	75	386.3 ± 24.9	99.91	70
	PVA/ACQDs	10	70	23.2	99.9	71
	GO/PVA/Zn(ii)	3.5	60	20.68	99.95	72
Cellulose and its derivatives	CA/PVC	3.5	75	18.56	93.3	81
	CA/PEG	5	80	20.46	—	82
	Surface treated CA	10	70	5.97	99.7	90
	CTA/SiO_2_	10	70	6.1	99.8	92
	CTA/Al_2_O_3_	10	70	6.7	99.8	93
	CTA/CNC	10	70	11.68	99.9	94
	Alkaline treated CTA/CNC	3	70	107.5	99.9	95
PAN	HPAN	3.5	30	24.4	99.9	108
	PVA/PAN	3.5	70	32.26	99.98	109
	HPAN-2	3.5	30	82	99.99	110
	NC-PAN	3.5	80	238.7	99.8	111
	PAN/PVDF	3.5	80	58	99.8	112
PA	PA-mCNT	3.5	70	104	99.99	120
	PA@GO	3.5	70	26.7	99.99	54
	PIP-NSO	3.5	70	50.54	99.99	56
	l-Lysine modified PA	5	70	30.5	99.6	121
	PEI/GO/PA	20	65	8.4	99.9	124
	PA-based TFC	3.5	70	95.1	99.1	125
Sulfonated block polymers	S-SEBS	3.5	70	22.87	98.4	128
	S-SEBS/PS	5	65	76.8	99.98	129
	S-SEBS/H302	5	65	9.3	99.9	130
	Nexar™	3.2	21	130	99.5	131

## 4. Cleaning contaminated PV polymer membranes

During extended operation of PV polymer-based membranes, the accumulation of organic fouling can diminish desalination efficiency. To mitigate this, chemical cleaning agents such as acids, bases and oxidizers are generally used, as well as the selection of appropriate membrane materials to significantly mitigate fouling problems.^132^ A study investigated the fouling resistance of AA-AMPS crosslinked PVA/PAN nanofiber TFCs was and PVA films exposed to Tween 20, sodium dodecylbenzenesulfonate (SDBS), and sodium alginate.^62^ The findings indicated that PVA effectively inhibited the formation of organic contaminants and salt scaling, with the fouling layer on the surface of the selective layer readily removable by water. Li *et al.* further enhanced membrane performance by grafting glycine-functionalized PVA onto polyamines (PD).^133^ This grafted PVA layer not only protects the PD layer but also maintains stability under both acidic and alkaline conditions, significantly improving the membrane's fouling resistance.

The incorporation of various functional materials (including metal–organic frameworks (MOFs) and carbon-based substances such as graphene and its derivatives) can enhance the chlorine and stain resistance of membranes. Ugur Nigiz *et al.* fabricated sodium alginate membranes enriched with GO and assessed their antibacterial properties.^134^ Upon direct bacterial exposure, the unmodified alginate membrane deteriorated, whereas the graphene-augmented membrane effectively suppressed bacterial proliferation on its surface. The addition of GO increased the hydrophilic functional groups, resulting in a flux enhancement from 1.63 kg m^−2^ h^−1^ to 4.89 kg m^−2^ h^−1^ at 40 °C; at 60 °C, the flux rose from 2.44 kg m^−2^ h^−1^ to 8.11 kg m^−2^ h^−1^, with the salt rejection rate remaining above 99.9%.

Sodium hypochlorite (NaClO) is a prevalent oxidizing cleaning agent that degrades polymer chains. To bolster the oxidation resistance of PVA-based membranes, Li *et al.* employed sodium periodate to eliminate the random 1–2 structure of hydroxyl groups in PVA, thereby enhancing the regularity and chlorination resistance of PVA.^135^ Concurrently, the fluorocarbon crosslinking agent FS-3100 was incorporated to further fortify the membrane's oxidation resistance. Subjecting the resultant composite membrane (PVA-FS/PVDF) to an oxidation resistance test using a 2000 mg per L NaClO solution demonstrated continuous performance over 168 h. When processing a 3.5% NaCl aqueous solution at 70 °C, the membrane achieved a water flux of 34 ± 1 kg m^−2^ h^−1^ and a NaCl removal rate of 99.93%. The membrane maintained a high salt rejection rate after exposure to acidic and alkaline conditions, underscoring the modified PVA's robust resistance to fouling and cleaning. Additionally, Li *et al.* reduced PVA's crystallinity and augmented the membrane's hydrophilicity by grafting polystyrene maleic anhydride (SMA) onto the modified PVA.^136^ The SMA-*g*-PVA/FS/PVDF composite membrane exhibited exceptional chlorine resistance, sustaining a high salt rejection rate of 99.9% after 384 h of exposure to a 2000 mg per L NaCl solution. This performance surpasses all previously reported PV and RO membranes, with a water flux of 44.5 ± 1.5 kg m^−2^ h^−1^ and a salt rejection rate of 99.93%. A long-term fouling and cleaning cycle experiment was conducted using humic acid at a concentration of 10 000 ppm to simulate organic pollutants. Post NaClO cleaning, the flux recovery rate reached 100%, indicating the prepared chlorine-resistant PV membrane's capability for sustainable seawater desalination.

**Fig. 13:**
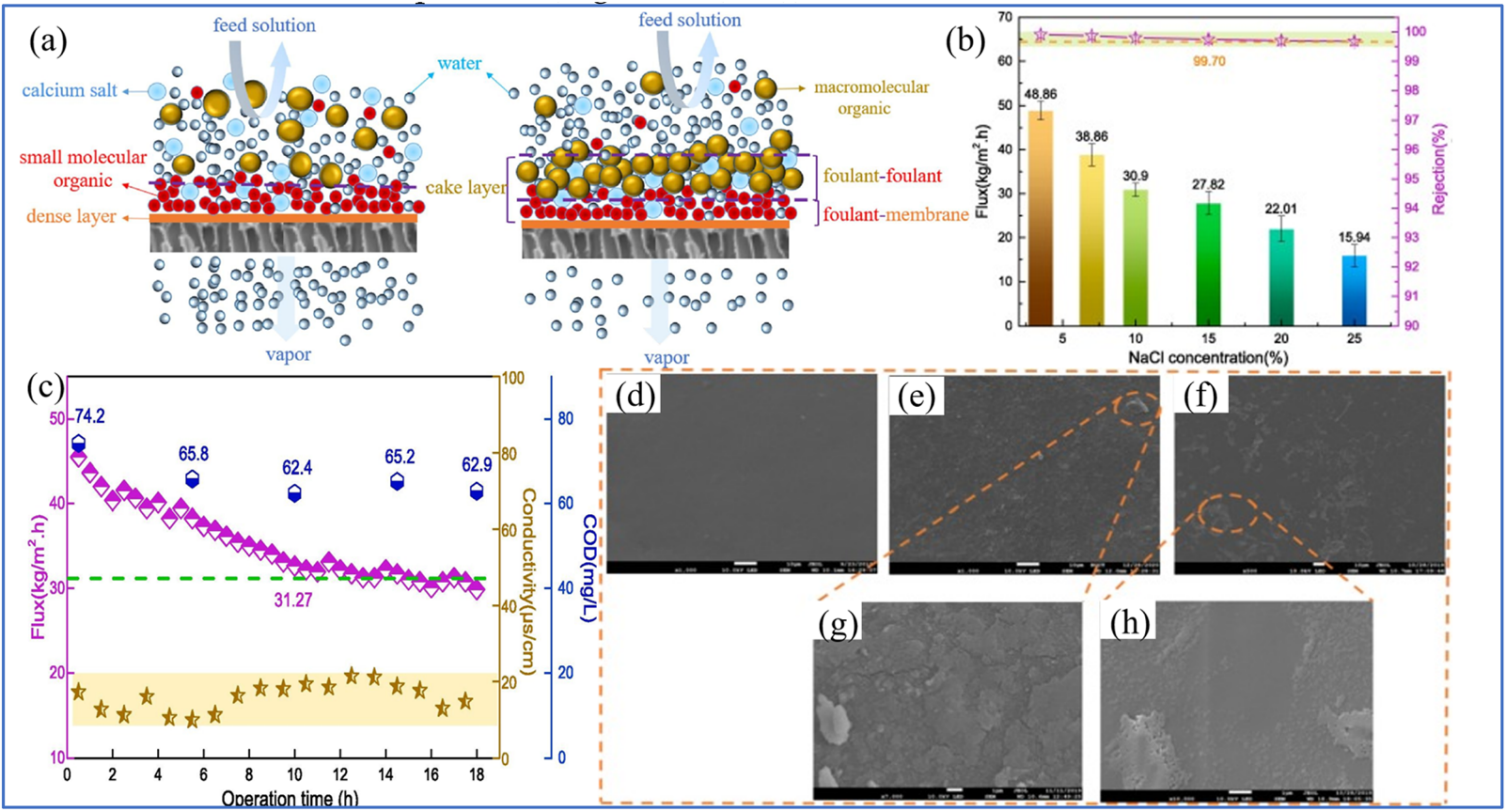
(a) Illustration of the two stages of membrane fouling. (b) Water flux and salt rejection of the PV membranes (c). Water flux and COD of the softened ROC PV membrane. (d)–(h) SEM images of (d) pristine PV membranes; (e) and (f) fouled softened ROC membranes; (g) and (h) cleaned membranes using NaOH.^137^ Reproduced from ref. 137 with permission from Elsevier, copyright 2021.

Li *et al.* pioneered the assessment of desalination efficacy and cleansing impact of a PV membrane on industrial RO concentrate (Fig. 13(a)).^137^ Utilizing a PVA-P(AA-AMPS)/PVDF composite membrane for RO concentrate treatment, they observed a sustained water flux of 48.9.27 kg m^−2^ h^−1^ at 3.5 wt% NaCl, with a water recovery rate of 98%, and the effluent quality complied with discharge regulations (Fig. 13(b)). The membrane achieved salt and chemical oxygen demand removal efficiencies of 99.90% and 90%, respectively and the membrane's water flux was restored to 98% of its original value post-cleaning (Fig. 13(c)). Optimal cleaning outcomes were realized with an initial 15-min treatment using 0.1 wt% NaOH, followed by a 15-min rinse with 0.02 wt% NaCl (Fig. 13(c)). The judicious selection of membrane materials, coupled with the incorporation of functional materials such as MOFs and carbon-based substances, can enhance the hydrophilicity of the membrane surface. This modification reduces the adhesion of organic pollutants to the membrane and bolsters its resistance to fouling. Furthermore, the establishment of a protective layer on the surface of the selective layer acts as a sacrificial barrier, safeguarding the integrity of the underlying protective film. Additionally, the design of membrane materials with robust tolerance to acid, alkali, and oxidant cleaning not only extends the service life of the membranes but also ensures their sustained and stable performance over extended periods (Fig. 13(d–h)).

## 5. Conclusions and perspective

PV is an emerging technology for seawater desalination that experiences minimal impact on water flux due to osmotic pressure, enabling the processing of high-salinity water to yield salt crystals and achieve zero emissions. However, the permeability of commercial PV desalination polymer membranes is relatively low. In recent years, the development of high-permeability PV desalination polymer membranes has the potential to reduce membrane area requirements, making them more cost-effective and appealing. Introducing polar functional groups on the surface of the selective layer or combining polymers with materials that facilitate water transport can enhance the membrane's hydrophilicity, provide additional pathways for water molecules, improve membrane selectivity and water flux, and strengthen the bonds with the support layer. Incorporating hydrophilic fillers or comonomers into IP to boost the surface hydrophilicity of the selective layer, establishing a protective layer on the surface of the selective layer, and designing membranes with robust cleaning resistance are effective strategies to ensure the long-term stable operation of the membrane. These approaches can extend the service life of the membrane material and improve its utilization rate. Moreover, modifying the support layer is a crucial method for enhancing PV membrane performance. By employing techniques such as blending and copolymerization to alter the support layer of polymer membranes, the mechanical properties of the separation layer can be improved, the mass transfer resistance of the support layer can be reduced, and the permeation flux of the PV membrane can be effectively increased.

Over the past five years, there has been extensive research on PV desalination polymer membranes. However, investigations into the fouling mechanisms of PV membranes remain comparatively scarce. The stain resistance and mechanisms underlying pollution generation in PV membranes warrant further exploration by the research community. In experimental settings, synthetic saltwater is predominantly utilized for testing purposes. Employing seawater for testing would offer a more accurate representation of PV membranes' practical functionality in seawater desalination, yet such studies are still infrequent. PV composite membranes are largely flat-sheet configurations, with other common forms such as hollow fiber, tubular, and spiral-wound membranes requiring additional research. Considering the potential of PV membranes in treating high-concentration saltwater, this technology is particularly well-suited for applications where reverse RO struggles, such as in the treatment of high-concentration saltwater, ROC wastewater, salt recovery, or zero-discharge scenarios.

## Author contributions

Writing – original draft preparation, Yufang Xu; investigation and validation, Chuanying Wang; project administration and funding acquisition, Lei Sang; reviewing and editing the manuscript, Qinghui Ling. All authors have read and agreed to the published version of the manuscript.

## Conflicts of interest

The authors declare no conflicts of interest.

## Data Availability

No new primary data were generated for this review. All data discussed or analyzed are drawn from the published research articles cited within the manuscript. Access to these data is governed by the policies and statements provided in the respective original publications.
